# Book Reviews

**Published:** 1826-05

**Authors:** 


### CRITICAL ANALYSIS

OF

### ENGLISH AND FOREIGN LITERATURE,

RELATIVE TO THE VARIOUS BRANCHES OF

### Medical Science.

Quæ laudanda forent, et quæ culpanda, vicissim

Illa, prius, cretâ; mox hæc, carbone, *notamus.*—PERSIUS.

### DIVISION I.

### ENGLISH.

ART. I.—**Transactions of the Medical and Physical Society of Calcutta.* Vol. I.—8vo. pp. xxvi. 409. Calcutta, 1825.

THE work, of which we are now about to render an account, is one of the most curious and interesting which has fallen within our notice for some time past; inasmuch as it is the first fruits of the labours of the Medical and Physical Society of Calcutta, a society instituted in the year 1823, and composed principally of the medical officers of his Majesty's and the India Company's service, whose objects and views are developed in a very sensible Preface, to which we shall presently call the reader's attention. We cannot however forbear, previously, from expressing our honest pride and satisfaction on receiving this solid testimonial of the zeal which animates our professional brethren in the East. We trust that this is only the precursor of many successive volumes; and that the healing art, as well as all those arts and sciences which tend to adorn life, and to render it more secure and more happy, may extend their influence, and finally overspread all those vast territories which have fallen under the government or protection of this country.

The volume before us contains thirty-three papers, on subjects chiefly medical and surgical, together with several shorter communications in the shape of an Appendix; with a list of the members of the Society, and an account of the donations towards the formation of a library and museum. It is illustrated with a few lithographic plates, and is printed in a very creditable manner, although the paper is but of an indifferent quality; which objection, however, applies to most that is manufactured in that country. This is, however, a very trifling matter. We might, with more reason, object to some few errors of composition, which we trace even in the Preface itself, and cannot but

* Although this work was published at Calcutta, we cannot well regard it as belonging to foreign literature, as all the contributors are of our own country: on this account, we have placed it among our English Reviews.

regret that the editors have not thought it necessary to explain the meaning of many of the Indian terms, with which European readers cannot be expected to be acquainted. Having mentioned these slight imperfections, we do not mean to insist upon them strongly, but proceed to lay before our readers an account of the origin of the Society, as detailed in the Preface to which we have before alluded: and we are the more disposed to enter thus abruptly into the subject, since the writer has anticipated almost all that we felt inclined to say relative to the utility of such associations:—

"The experience of modern times has established the advantage of miscellaneous collections, in which the unconnected observations of different individuals are brought together and preserved; and the periodical publications, which form so prominent a feature in the literature of the age, supply valuable materials to permanent compilation, and foster and keep alive the spirit of research.

"The benefits of occasional publication are in no case more evident than in the science of medicine. Amidst the varied opportunities of observation which its unbounded sphere of action developes, many circumstances of a peculiar character, many conclusions of wide applicability, must occur to its individual practitioners. The experience of its professors is the common property of the profession; but incidental reflection may be too brief for formal record,—a solitary fact too unsafe a base for generalisation; and neither the one nor the other would, therefore, be communicated to the world, unless there existed some unpretending repository, in which they might be registered for further verification or correction. Periodical publications are to us what the tables in the temple of Æsculapius were to his ministers, with all the advantages derivable from the improved nature of the medium, and the more justly grounded doctrines of modern practice.

"If these obvious advantages have led to the multiplication of medical journals in Europe, they constitute motives equally powerful for the introduction of similar publications in Asia; but there are various considerations calculated to enhance their utility in this country, arising from local peculiarities, and the circumstances under which the professors of medicine are here placed." (P. i. ii.)

Among these circumstances, the influence of climate, food, &c. upon disease is noticed, as well as the advantage to be derived from the introduction of new medicinal agents; among which the pomegranate bark, the madar bark, and the croton oil, are particularly mentioned.

The following passage is so just and so candid, that we cannot forbear to quote it, since it may serve as a reply to those who may be inclined to think that this volume contains less of novelty than might have been expected.

"Whatever advantages may be realised from inquiries thus favourably instituted, will be shared by us with our brethren of the West; but some benefits may be expected from an improved circulation of useful information amongst ourselves, which may be regarded as exclusively our own. The situation of medical men in India labours under many discouraging peculiarities: with the exception of the few among them who are resident at the principal cities and civil stations, they are scattered far apart from each other, over a vast extent of country; they hear and see nothing of the stir of science, and catch but indistinct and partial glimpses of the advancement of knowledge. Their limited means and frequent removals put it out of their power to provide themselves with regular and expensive supplies of books, and there are no public libraries beyond the limits of the Presidencies: they can derive, therefore, little benefit from the recorded experience of others; and have rarely an opportunity of confirming or correcting their own by that of an associate, a rival, or a friend. Their scene of action is too restricted, and too entirely their own, to admit of emulation or ambition; and the official Reports, in which alone their practice is commemorated, are too much matters of form and routine, to merit or attract animadversion. So situated, it is not wonderful that their zeal dies within them: no man is independent of external stimulus, and the surest method of annihilating energy is to leave it to prey upon itself.

"Considerations of this tenor, affecting the prosperity and credit of the profession in India, rather than aspiring to a competition with its distinguished and veteran combinations in Europe, led to the formation of the Medical and Physical Society of Calcutta. Publication was only a possible contingency: the immediate object was to give a concentric impulse to the detached members of the service, and to afford them augmented facility of information, as well as a new excitement to emulative exertion. They were therefore invited to contribute, without restraint, the results of their inquiries and observations to the associated members resident in Calcutta; and the proceedings of the meetings at the Presidency were, in return, to be regularly communicated to the non-resident members; the liberality of the government of Bengal enabling the Society, in its infancy, to maintain these communications, by exempting them from postage for the first year after its institution. The result of the appeal fully justified expectation, and demonstrated that means only were wanting to animate the energies of the service. A very considerable portion of the establishment of Bengal, and several members of that of Bombay, were speedily enrolled in the lists of the Society, including the Medical Boards of both Presidencies. The Society has also received proofs of a friendly interest taken in its proceedings by the Medical Board and service of Madras, although it hitherto enumerates but few of the members of that branch of the establishment. The general support, however, it has received, would have justified the Society in assuming the denomination of the Medical Society of India, had not an appellation been previously adopted, with reference to the immediate seat of its institution." (P. v.—vii.)

"In conclusion, the Society feel confident that the volume will be received with the candid judgment of medical men, and will be recognised as an unequivocal testimony of undiminished interest in the credit and duties of the profession." We, in our turn, feel confident that these expectations will not be disappointed; and we cannot but take this opportunity of noticing, with an exultation derived from an ardent attachment to our profession, the enthusiasm which appears to animate all its professors, even under the most discouraging circumstances; an enthusiasm arising, we firmly believe, from the purest and most honourable motives. This we conceive more particularly applies to those medical men engaged in the different departments of the public service, who in general have little else to hope for, from the exercise of their profession, than the consciousness of having performed a most sacred duty, and of having contributed their best endeavours towards the relief of suffering humanity.

1. The first paper in the volume is an account of the *Kushta, or Leprosy, as known to the Hindus*, by H. H. Wilson, Esq. —Mr. Wilson appears to have paid much attention to the medical literature of the Hindus, of which he promises to give a comprehensive sketch at a subsequent period. His object, in the above Memoir, is to point out the principal Indian writers on Leprosy, with their opinions as to the causes, seat, and nature of the disease, as well as its mode of treatment. We shall not enter into this inquiry, since it does not appear to us that any practical advantages are to be derived from the minute distinctions entered into by these writers, who enumerate eighteen different kinds of leprosy, seven great or principal, and eleven of minor importance. They also tell us that the disease affects the seven essential parts of the body, besides the three humours, wind, phlegm, and bile; the seven essential parts of the body being lymph, blood, flesh, adeps, bone, marrow, and semen. Neither can it be of much advantage to detail the various external applications recommended by them as remedies, considering that they are composed of the most heterogeneous and multifarious ingredients, some of them containing more than thirty articles, and in that respect resembling those costly compounds formerly so much in vogue in Europe. The belief of the contagious properties and hereditary transmission of the disease, seems to be universal among the native practitioners.

Mr. Wilson devotes a page or two in combatting the arrangement of this disease, as usually met with in English writers, and especially in Dr. Good's classification; and we are inclined to think that there is some justice in the objections which he has urged upon this point.

2. Of the second paper we shall do little more than give the title. It does not appear, from the account of the author, that the tree has ever been employed in medicine, and it is only proposed to be tried as a substitute for the American sassafras bark. It is a *description of the Tree which produces the Nipal Camphor Wood and Sassafras Bark;* by N. WALLICH, M. and Phil. D. It is a species of Laurus, differing both from the L. camphorifera of Kæmpfer, from the L. sassafras of North America, and the L. parthenoxylon, of which an account was published in the Malayan Miscellanies, and which is described by ROXBURGH in his manuscript Flora Indica, by the name of Laura porrecta. It appears that the species described by Dr. Wallich grows but sparingly in the mountains of Nipaul, at an elevation of between six and eight thousand feet above the level of the sea.

3. *Case of fatal Effects of the Bite of a Venomous Snake.* By P. BRETON, Esq.

4. *Case of Recovery from a Wound in the Stomach, inflicted by a Pistol-ball.* By P. BRETON, Esq.

This case being extraordinary, and in our estimation any thing but convincing, we give it, without abridgment, in the author's own words, that our readers may form their own judgment.

"A trooper of the irregular cavalry, attached to the Ramghur corps, in a fit of despondency, and to avenge himself of the Risalehdar, by whom he had been reproved for negligence of duty, formed the resolution, on the 30th of April, 1819, to commit suicide.

"In the evening, about dusk, he took his holster pistol and three ball cartilages, and proceeded from the fort of Sembhelpur to the bed of the Mahánadi River, where he intended to effect his purpose. His woman, seeing him in a disturbed state of mind, followed him at a short distance. Observing him load his pistol, and directing it towards himself, she ran and seized his arm, and endeavoured to force the pistol from his hand. In the struggle, the pistol was fired, and the ball passed through the body of the woman, and killed her on the spot. The trooper again loaded, and shot himself in the pit of the stomach, the ball lodging in the left side of the lumbar vertebræ.

"Having failed in his attempt to destroy himself, and the third ball cartilage with which he had provided himself being lost when he fell on the ground, by the shock from the ball, he returned, without any assistance, to the fort, and communicated every thing that had happened. The corpse of the poor woman was immediately sent for, and brought to me, together with the trooper who had caused her death.

"On examining his wound, I found that the ball had entered the epigastric region, and was perceptible to the feel in the left side of the loins. I immediately extracted it, and closed the incision with adhesive plaster. Conceiving that the stomach was penetrated by the ball, I considered the case a hopeless one, and reported the wound to be mortal. The following morning, the man had recovered his senses, so as to give a distinct and rational account of all the circumstances which led to the commission of the rash act; and the wound manifested such appearances as left no doubt in my mind of the stomach being perforated.

"My attention was, therefore, directed to uniting the anterior wound with all possible speed, and to prevent the introduction into the stomach of any more food than was absolutely necessary to support life. To my astonishment, the wound quickly united, and the serous discharge from the stomach gradually ceased. When union had taken place in the anterior wound, I was informed by the native doctor that, when food was taken into the stomach, particles now and then escaped from the wound in the loins, where the ball lodged. To convince myself of the truth of this assertion, I gave the trooper a draught of water to drink; and, almost immediately afterwards, I distinctly saw a small quantity of it trickle through the wound. I repeated this two or three times, and was satisfied that there was a communication between the stomach and the wound in the loins. Superficial dressings were continued, and rigid abstinence enjoined. In the course of about a month and a half, the wounds healed, and the man recovered, and was afterwards tried by a general court-martial for the crime of murder.

"It is remarkable that, in this case of perforation of the stomach by a pistol-ball, very little pain and uneasiness were expressed by the patient, and symptoms of irritation were not at any period manifested. Neither was thirst complained of, nor were the natural functions of the body in any considerable degree disturbed.

"Mr. G. King, surgeon of the 4th native regiment, who was at Sembhelpur at the time of the occurrence of the event, and was present when the trooper was first brought to me, can bear testimony to the nature of the wound, of which the above is a description." (P. 59—61.)

### 5. *On the Exhibition of Phosphorus in Cholera Morbus.* By John Adam, M.D.

This paper is ushered in with the following remarks:—

"In an abstract of cases treated in the general hospital, which I had the honour to present to the Society at our last meeting, I described the epidemic cholera as assuming a severe form, and proving extremely fatal among a detachment of H. C.'s recruits recently arrived from England. The total inefficacy of the means employed in those cases, induced me to propose some remedies of a powerfully stimulating nature, whose application may be considered in a manner new to British practice. Phosphorus, to which I more particularly alluded, has not a place in any of our Dispensatories, and the trials which have been made with it, as far as I am acquainted, are confined to the physicians of the continent, especially of Germany and Poland. From the accounts which some of these have furnished of its effects in disease, it appears to be a very powerful medical agent; but the supposed poisonous qualities of the drug, and its singular susceptibility of combustion, have combined to prevent its more extended use; and the most tragical consequences seem to have been apprehended, both by patient and practitioner, from the presence of even a minute quantity in the stomach. How far, on the one hand, such fears may be well grounded, or, on the other, the medical virtues it is declared to possess, be not hastily assumed, from a limited and partial observation of its effects, I am not prepared to determine. All the information which I have been able to procure on the subject, is contained in Hooper's Medical Dictionary, under the article Phosphorus; and, from a perusal of the statement there given, I was satisfied that it was deserving of a trial in cholera morbus, and I resolved on administering it in the first case which occurred. Before detailing the results of my own experience with this remedy, I shall here make a few extracts from the above work, in order to show the condition in which the patients are represented to have been at the period of its exhibition, and the encouragement thereby held out for extending its use, by analogy, to cholera." (P. 62, 63.)

These extracts we omit, and proceed to the history of some cases in which the phosphorus was employed by our author: they are three in number, all Europeans, stout young men, belonging to the 13th regiment. We shall detail the first of these cases in Dr. Adam's own words:—

"Michael Woden, H. M.'s 13th Light Infantry Regiment, twenty-three years of age, an uncommonly stout muscular man, was admitted on the 24th May, about half-past four A.M., in the second stage of cholera morbus; and had one scruple of calomel, with two grains of opium, which were speedily rejected. At the morning visit at six o'clock, he was in a state of great exhaustion. Aspect livid and languid; pulse barely perceptible; skin not so moist as usual; cramps in both extremities; two or three stools since taking the medicine, of the peculiar white watery kind characteristic of cholera. Two grains of opium and ten of camphor were administered, with an enema of two drachms of tincture of opium. In the course of the day, no improvement taking place, phosphorus was given in the dose of two grains, repeated. It was taken in the solid form, rolled up in small portions of soft bread. No particular effect was observed at the time of swallowing it, but it was retained on the stomach. At the evening visit, the pulse was quite gone, skin cold, and he lay in a sort of stupor, as if asleep, or in articulo mortis. Three grains of phosphorus were then ordered, with bottles of warm water to the feet and legs, and a mustard cataplasm to the stomach. At nine P.M. he looked rather better, but otherwise the symptoms were not more favourable; the whole surface cold and covered with moisture, and thirst extreme. The phosphorus was repeated, in the dose of three grains. On the 25th, next morning, he was not only improved in his looks, but appeared altogether better than was anticipated. Face and hands still livid and cold; pulse perceptible, but excessively feeble; tongue white; had no complaint of pain, but was drowsy, as if under the influence of the opiates. The phosphorus was prescribed in the dose of two grains, and ordered to be given again at ten o'clock, in the dose of one and a half grain, in small divided pieces, unless the surface previously became warm. No relief ensued, and he died at one P.M.

"*Appearances on dissection of the body.*—The post-mortem examination was made principally with a view of ascertaining the effects of phosphorus on the stomach and intestines. The remedy had been exhibited at first in one piece, rolled up in soft bread slightly moistened, so as to form a sort of paste enveloping the phosphorus; but latterly it was given in divided fragments, also rolled up in bread, but so slightly coherent as to yield readily to the action of the stomach, and allow the phosphorus to come into apposition with its internal coat. The intestines externally presented a reddish appearance, as if slightly inflamed, and there was some congestion in the veins of the stomach. On laying open the stomach, a dark reddish colour was observed towards the cardiac orifice, and a greater portion of mucus was effused than is commonly met with in cases of the disease. A large piece of phosphorus, completely enveloped in the bread used as its vehicle, was found near the pylorus, lying in the form of a ball. Beyond this, the internal coat of the intestines was covered with a mucous or puriform effusion, resembling the matter of gonorrhœa or tragacanth paste, and inflamed, as is observed in those who die in the advanced stage of cholera. The other viscera appeared sound." (P. 65—67.)

From these histories, and the accompanying dissections, we learn only that phosphorus does not produce any deleterious effect on the system, when taken in pretty large doses; for we cannot allow ourselves to believe that any advantage was derived from its exhibition; and therefore the cases related by our author only go to prove the safety and practicability of submitting the powers of the remedy to the test of further experience. To those who are inclined to make trial of its efficacy, the following observations will be valuable:—

"With regard to the form of administering phosphorus, that of solution in ether, occasionally adopted by the German physicians quoted above, appears the preferable mode. The stimulating properties of the menstruum will tend to aid its operation; while the fluid, by being diffused over a larger portion of the stomach, will prove the more efficient. When I ventured on prescribing this remedy in cholera, I wished to make trial of the solution, but could not obtain at the time an ether sufficiently concentrated to prepare it. I have not since repeated the experiment; although, with the assistance of heat, there can be no difficulty, I apprehend, in accomplishing this object. The solution in expressed oil does not appear well adapted to the purpose in view; but it may be deserving of inquiry whether the volatile oils, as turpentine, would not furnish even a more convenient and efficacious vehicle than the ethers.

"Before concluding these remarks, I must observe, that the exhibition of phosphorus is admissible only in the second stage of cholera, or during that period of exhaustion and collapse which succeeds the first attack. At the very commencement of the disorder, when much gastric irritation prevails, and in the last or febrile stage, direct stimulation, as a general plan, is obviously contra-indicated. Still, in the occasional sinkings which occur after the operation of purgatives, even in this stage, the phosphoric solution might be used with advantage, if greatly diluted. It is not easy, however, to lay down fixed rules for the treatment of this disorder. The varying character it displays in its progress requires a corresponding variation in the remedies employed, and the utmost vigilance to be exercised on the part of the practitioner, in order that he may meet the symptoms as they arise. It is to be lamented, indeed, that all the skill and care he can bestow will prove too often unavailing. The disorder, in the majority of instances, continues to hold on its course unchanged; and, though protracted for a longer or shorter time, ultimately bears its victim to the grave, in defiance of every remedy that reason and experience combined can suggest." (P. 72, 73.)

6. *Notice on Oil in the Blood.* By JOHN ADAM, M.D.

7. *On the Madár, and its Medical Uses.* By G. PLAYFAIR, Esq.

The author presents us, in this paper, with a botanical description of the plant in question, the method of preparing the root for internal administration, and then enumerates the diseases in which it has been found efficacious; concluding with extracts and copies of correspondence with gentlemen of the profession, in corroboration of the beneficial effects said to result from the use of the madár as a medicine.

Mr. Playfair gives the following account of the reasons that first induced him to make trial of the madár root:—

"In the year 1811, I was a good deal engaged in an investigation, the object of which was to ascertain the medical properties of plants used by the native physicians; and amongst others I met with the madár, as one item in a recipe for the cure of 'Jezam.' The proportion was very small, and the ingredients of the formula numerous; but, as the use of it was evidently attended with some benefit, I determined to institute an inquiry into the virtues of each article separately; but I was under the necessity of proceeding with great caution, as, in every other work which I consulted, the madár, or *aak*, was mentioned as an active poison. Having discovered its effects in the above-mentioned complaint, and reasoning from analogy, I proceeded to try its effects in other diseases.

"After mature consideration and conviction of the efficacy of this powerful agent, I communicated the discovery to several gentlemen high in the profession, who assisted me in extending the scale of my experiments. In 1814, I addressed the Medical Board on the subject, and solicited that the discovery might be honoured with their patronage.

"In addressing the Medical Board, my object, of course, was that, by their sanction and influence, a much more extended trial might be made than could be effected by the exertions of an individual; and I expressed a hope that they would be induced to sanction its use in some of the hospitals, where a greater variety of diseases were to be met with than could be expected in my limited practice. At the request of the Board, I forwarded a quantity of the medicine properly prepared, of which they were pleased to order a trial to be made in the general hospital at Calcutta. In August, 1814, I was informed by the Medical Board that the trial had been made, and that, as far as the experiments had then been carried, they were considered encouraging to a further perseverance. I was desired to send a further supply of the medicine, as the quantity I had forwarded had been too small to produce any decisive result. This, of course, was complied with immediately; and in March, 1815, I was honoured by receiving copies of a letter and report, which had been given in by the surgeon of the general hospital. The Medical Board concluded, that, although the experiments were not very favourable to the opinion that the madár was possessed of extraordinary medical virtues, yet they observed, no ground was afforded for any very positive inference on the subject; and, as the trials had been confined to one disease only, the efficacy of the medicine still remained to be ascertained by more diversified experiments in a variety of other affections." (P. 78, 79.)

It appears, however, that it had only been employed in syphilis, and that in a very limited number of cases; the doses, also, had not been properly regulated, and therefore the results were not so favourable as might have been expected. This led to further explanations with the Medical Board, but it does not appear that any fresh evidence, as to the efficacy of the madár, emanated from that quarter.

Some experiments made in the native hospital at Benares were extremely favourable, as well as in several European hospitals, as will presently appear. The author adds, that, since the year 1811, he has administered it to a variety of patients, with the happiest results; and every year's experience has added fresh conviction to his mind of the intrinsic value of the medicine.

The madár root is said to be a powerful tonic and alterative, stimulant and deobstruent, and in combination with opium it is sudorific. The complaints in which it has been given are syphilis, lepra, and other cutaneous eruptions; dropsy, rheumatism, glandular obstructions, tape-worm, and intermittents. It has been found efficacious also in the lupus, which is very common among the natives of India.

The cases in which the curative powers of the madár have been most strikingly exemplified, next follow. The first case is one of a severe herpetic affection in a native, cured by the madár root, in doses of five grains twice a-day. The next is related by one of our author's correspondents: it is a case of leprosy, which had existed five months, and had resisted every remedy previously employed. It was given in the quantity of three grains three times a-day, and gradually increased. In about six or eight days, the complaint was evidently on the decline; in about fourteen, not above one half of the original diseased surface remained, and the itching and pain were entirely gone. The complaint was, after about six weeks, quite removed, the skin smooth and level. The patient never complained of any uneasiness, although latterly he had taken to the extent of thirty grains daily. The same correspondent relates two other histories, one of remittent fever in a child, the other of pulmonary complaint in an adult, where this medicine was eminently successful. It was also used as an external application in ill-conditioned sores, with the happiest effect.

Other professional gentlemen testify very strongly also in favour of this new remedy, chiefly in leprosy, syphilis, and tape-worm.

Our author sums up the general results of his communications and experiments in the following words:—

"It will be observed, that I have allowed the merits of this medicine to rest almost entirely on the evidence of members of the profession, whose opinions must be considered of much more value than my own, and less liable to the imputation of partiality, or generalising too fondly from what might have been considered doubtful premises. I shall, therefore, conclude with the fervent hope that the madár will continue to attract the attention of the profession, and by offering my sincere thanks to those gentlemen who have so essentially contributed to enable me to lay it before the public in its present state." (P. 102.)

The two next papers we pass over entirely. The first relates to *the appearance of Locusts in the Doab,* by G. Playfair, Esq.; and the second to a *Luminous appearance of the Ocean,* by J. Henderson, Esq. They are not without their interest to the naturalist and man of science; but our object is more especially the description of professional subjects, and we have still a large portion of the volume to examine.

We pass on, therefore, to the tenth article, which is a *Case of Fungoid Disease,* by R. N. Burnard. The patient was a Bungalee, aged thirty-three; the disease was situated in the knee, and had existed for a twelvemonth. The tumor was large, and ulcerated to some extent; the ligamentum patellæ was absorbed, the muscles of the thigh wasted, and the whole body extremely emaciated. There was hectic fever present, though neither much cough nor expectoration. As, however, the absorbent glands were not enlarged, and there did not appear the least vestige of a similar disease in any other part, amputation was proposed and performed. This man recovered perfectly from the operation, though whether his recovery will be permanent or not remains yet to be seen. From this case Mr. Burnard draws the following conclusions:—

"1st. That a malignant disease may in some instances remain for a long time local, and exhaust the system simply by irritation. 2dly. That malignant diseases do not always extend by absorption, as in this case there was no affection of any part of the absorbent system. 3dly. That, where the disease is confined to one part, and there is no evidence of similar disease in the viscera or in the absorbent system, no state of emaciation or debility ought to prevent the removal of the part by operation, if practicable.

"It should have been noticed, that, previously to the operation, the colour of several parts of this man's body was nearly removed, and his face, hands, and feet, instead of the natural olive colour, were almost white; but, as he regained strength, the natural colour returned, and they are now uniform." (P. 115, 116.)

Article 13th is an *Essay on Delirium Tremens*, by G. Playfair, Esq. We should not, perhaps, have been induced to notice this paper, excepting that it confirms the line of practice adopted in Europe for the cure of this complaint, and contains a very satisfactory account of the disease, its causes, and method of treatment.

14. *On Strychnos Nux Vomica.* By T. E. Baker, Esq.

This gentleman was induced to direct his inquiries into the effects of the above medicine, in consequence of an accident which happened to a servant at the Honourable Company's stud depôt.

"A man, named Ram Serdek, was carrying an umbrella for Lieut. Hailes, an officer of the stud, when he suddenly fell down in a fit: he appeared stiff and lifeless, and, when raised up, stood upon his heels, with the toes and feet turned upwards; the eyes remained open, but fixed; and the jaws were so firmly closed, that it was some time before they could be separated, in order to pour down some spirits of hartshorn and water. He recovered in the course of a few minutes, and soon after vomited.

'When I saw the man, he was perfectly recovered, except from the effect of the bruises which he had received in his fall. On inquiring into the cause of the fit, he said it was owing to his having taken the *kuchila* (nux vomica) that morning, and not having eaten any thing before or afterwards, having merely drank a little milk. He took the medicine at seven o'clock, and had the fit two hours after: he had been taking it regularly for four months. He began with the eighth of a nut, the usual dose, which was always taken morning and evening, immediately after eating his meals: the quantity was gradually increased, and, for the last month, he had taken a whole nut morning and evening, which quantity must never be exceeded. A nut weighs about twenty grains." (P. 138, 139.)

The nux vomica is also used by the natives as a preventive of hydrophobia. When a person is bitten by a dog supposed to be rabid, they give one-eighth of a nut, morning and evening, for seventy-one days; and a nut, roasted and mixed with linseed oil, is applied to the bitten parts. A native doctor told the author that twelve people, bitten by mad dogs, all escaped the disease by using this remedy. We are also told that, when hydrophobia has taken place, it is sometimes used with success.

The only other complaints for which the nux vomica is used are those anasarcous swellings of the lower extremities which so frequently occur after fever. It is always given either immediately before or after meals, otherwise it is apt to produce giddiness. The Buceros (hornbill) eats it at all times

Articles 17th, 18th, and 19th, are papers on the *Dracunculus*, by J. Bird, Esq. by Dr. Kennedy, and by G. Smyttan, M.D. Though occupying a considerable number of pages, these observations contain but little of novelty, either as to the description of the worm, the causes of its invasion, or the method of cure. They principally go to confirm what has been previously said upon the subject by Mr. Paton, Mr. Scott, and Dr. Chisholm.

Want of space obliges us to pass over several articles, some of great local interest, no doubt, and others very excellent examples of the diseases of which they treat.

The next paper which arrests our attention is the *Case of a singular Tumor in a Native*, by J. Adam, M.D.

"The size and singular form of the tumor, together with the appearance of his body generally, studded over with small berry-like elevations, immediately attracted my attention; and I was glad to avail myself of the opportunity of becoming acquainted with a variety of cutaneous disease which seemed in a great measure new, and whose investigation, I conceived, might be interesting to the Society and the profession at large. From some resemblance which the parts bore to the diseased surface in elephantiasis, the effect produced on the eye of the beholder was far from agreeable. A dark wrinkled mass, in colour and texture not unlike the skin of a toad's back, hung down over the neck, in longitudinal folds, extending from above the right ear to the middle of the pectoral muscle. It was attached, at the upper extremity, to the loose scalp surrounding the ear, and was there comparatively narrow; but, as the tumor descended, its base embraced a wider range, and included, towards the chin, the greater portion of the integuments covering the superior and lateral aspect of the neck. At the angle of the jaw, it appeared also to penetrate more deeply, and to be not wholly unconnected with the blood-vessels and nerves lying in that situation. When held up, outstretched, it was seen to be divided by an irregular perpendicular line, with lateral ramifications, into two unequal portions; the largest being situated beyond the ear, and the smaller forming the termination at the chin. In this position it measured rather more than one foot in its longest diameter, and eleven inches in its shortest. Its thickness at the lowest part might have been about three inches; but higher up, towards the ear, it did not exceed one and a half. To the feel it was doughy and inelastic, and was not endued with any great degree of sensibility; for, when pinched rather freely, no uneasiness whatever was produced, except at particular spots, which were indurated, and evidently proceeded from absorbent glands involved in the general mass. In the portion of the tumor situated next the chin, one of these had attained the size of a duck's egg, and was exquisitely tender to the touch; but in the other parts he could bear the roughest handling without complaint. There were several small tumors in the sound scalp behind the large mass, which appeared to partake of the same glandular character as the one alluded to, and differed in many respects from the tubercles dispersed over the surface. This difference was indicated both by the greater hardness and sensibility of the former, previous to the removal of the tumor from the neck, and the enlargement and inflammatory action which they underwent subsequently to it. All over the surface, the skin was elevated into globular tubercles, which were small on the face and forehead, but increased in size on the trunk and lower extremities. The largest of these was situated a little above the right nipple, or midway between it and the axilla, and did not exceed an ordinary walnut. There were several large ones also on the back and lower extremities; but, on the arms and hands, they were not only smaller, but less numerous and distinct. All of these, with exception of the large mass in the neck, were disposed to be sessile, and were attached to the skin by a base occupying, in general, about a third of the sphere which they represented. When grasped between the fingers and thumb, they were found not so soft and yielding as might have been expected; and it seemed that, while the skin itself had lost its elasticity, some change had been effected in the texture immediately underneath, by which this quality was increased in it, and a power of resistance added that did not belong to the natural and healthy condition of the parts. In the interstices of the tubercles, no apparent alteration had taken place in the skin, which was, in point of colour, not darker than is usually observed among the lower order of natives. The large tumor alone, as mentioned above, was deeper in its shade than any other part of the exposed surface." (P. 299—302.)

The man was muscular, and generally healthy; his chief annoyance was the weight of the tumor, and a pricking and tingling sensation in it. He appeared about twenty-two or twenty-three years of age, and the tumor had existed about half that period. This man was very desirous that something should be done for the removal of the tumor; but, as the affection appeared not to be local, as far as regarded the cuticular texture, the author thought it could not be successfully attacked but through the medium of the system. With this view, the liquor arsenicalis was prescribed. After two or three weeks, the tumor became painful, and the tingling sensation was increased; febrile irritation also came on, and the arsenic was consequently omitted. When the feverish symptoms had subsided, it did not appear that any alteration had been produced in the tumor, and he became more solicitous than ever that the diseased mass should be cut off. In compliance, therefore, with his wishes, this was done.

"With the assistance of my friends, Mr. Hamilton and Dr. Mouat, of H. M.'s 13th Light Infantry, (who both took a particular interest in the case,) I removed the larger portion of the tumor by a simple incision in the course of the apparent line, commencing a little above the ear, and terminating at the most depending part, where it rested in contact with the breast. Contrary to expectation, the hemorrhage was profuse, and we found it necessary to secure five or six pretty large vessels before applying the dressings. A few stitches were used to bring the retracted edges together, and adhesive straps, with the usual pledget, placed over all. Suppuration took place in the most kindly manner in the whole line of the wound, and after five or six weeks the sore was completely cicatrised. A considerable portion of the tumor next the chin still remained adherent by its base, but was productive of no inconvenience; and the patient repeatedly declared the relief he experienced from the operation. Had the removal of the whole tumor been determined on, it must have proved a work of some difficulty, and not altogether exempt from danger, as its connexions at the angle of the jaw were pretty deeply seated; and the attachment to the neck was so extensive, that a large wound must have been the result, with such subsequent irritation as would have proved injurious to the object in view. The excised portion weighed between two and three pounds: it was composed of elongated skin and condensed cellular membrane, but so altered in structure as to be scarcely recognisable. Diseased absorbent vessels appeared to be mixed with the hardened adipose substance, and the whole resembled not a little the thickened state of the integuments so frequently met with around the knee-joint, in cases of white swelling.

"After the wound was closed, I wished to try the effects of iodine, whose powers in removing various tubercular affections have been so highly extolled of late; and I began by administering the tincture in the dose recommended. But my patient, having now attained the object which brought him to the Presidency, was desirous of returning to his home. He ceased his attendance for a day or two; and, on inquiry being made, it was found that he had departed for the country, without giving the slightest intimation; and I heard no more of him afterwards." (P. 305—307.)

The two last Essays contained in the volume relate to the *Worm found in the Eye of the Horse;* the first is by P. Breton, Esq., the second by W. Twining, Esq. The first paper commences thus:—

"At the last meeting of the Medical Society, I was desirous to bear testimony to the fact asserted by a member present, of the existence of the disease termed 'worm in the eye of horses,' but was restrained by a wish to hear the sentiments of the other members, better informed than myself on this singular malady.

"If an opinion can be formed from the silence of the members who were present when the appeal was made to the Society for corroboration of the fact stated, the existence of the malady was not generally believed. The removal, however, of all doubt of the presence of the worm in the eye of horses from actual demonstration, enables me to write with confidence in confirmation of the assertion.

" Several instances of the case have fallen under my own observation, and in more than one instance success attended me in the operation of extraction. The worm which I had an opportunity of examining, and preserving some time in spirit of wine, resembled very much the appearance of a piece of very narrow tape. It was upwards of an inch in length, was soft in texture, and it died in a few seconds after its extraction. Whilst swimming in the aqueous humour of the anterior chamber of the eye, it appeared of a white colour and flat, and its motion was precisely that of a leech in water. Its motion appeared to me to be incessant. I watched its action upwards of an hour, without observing the slightest diminution of it; and the natives seem to think that it does not fasten and rest itself as a leech does in a bottle of water, but continues in constant motion. This, however, I conceive to be mere hypothesis; for their observations, in general, on subtle points are not such as to warrant belief in the accuracy of their opinions.

" The motion of the worm in the aqueous humor seems by degrees to excite a slight irritation. The aqueous humour gradually assumes a milky appearance, slight inflammation of the tunica conjunctiva ensues, and water begins to trickle from the eye. If the worm remain, inflammation of the iris arises, the aqueous humour becomes turbid, and the coagulable lymph of the blood begins to manifest itself within the laminæ of the transparent cornea. This affection continues till the opacity of the cornea is completed. The inflammation then seems gradually to subside; but whether from the death of the worm at this stage of the disease, I had no opportunity of ascertaining." (P. 337, 338.)

Privation of sight is the invariable concomitant of this worm, if not removed in time; and extraction by incision into the cornea is the only effectual remedy. Horses troubled with a worm in the eye are also subject to weakness in the loins: this is the universal belief of the natives, and receives confirmation from the following remarks of Mr. Grelies, veterinary surgeon of H. M.'s 25th Light Dragoons.

" This wonderful phenomenon, or production in the animal economy of the horse, I will not presume to explain; for I have witnessed but one case, which was on my first arrival, and being under some fear, from the very ferocious description I had received of the animal, I would not venture to operate, unless he was previously thrown; in consequence of which I was not successful, although I made two very extensive incisions immediately over the worm, as he moved on the surface; but, from the position of the horse's head on the ground, I ought to have foreseen the impossibility of the worm's escaping with the watery humour, which is the object of incision; for, when the head is confined to the ground, the water naturally gravitates to the posterior chamber of the eye, consequently neither water nor worm can escape by incising in that posture. It will be needless to add, that the successful mode of operation is to insert the lancet while the horse is standing: if possible, the incision should be made while the worm is floating on the surface of the eye, and a little beneath it, by which it will immediately pass out with the water. Some care is required not to make the incision too extensive, as the crystalline lens may also escape, which would cause immediate blindness.

"I have heard that mercurial applications to the eye will destroy the worm, which being absorbed, the vision will not be impaired. However extraordinary this mode of cure may appear, it is not so much so as the disease; and I conceive it worthy a trial, as the texture of the eye would not be so much deranged as by incising.

"I have been informed by many gentlemen, that weakness in the loins frequently succeeds the extraction of the worm, which I believe; but I very much doubt whether the one is in consequence of the other. It is possible that a relaxation of the nervous system may, however remotely, cause the worm in the eye, as it is a disease confined to hot climates; and, as I firmly believe the weakness in the loins to be some paralytic affection of the spinal marrow or nerves, so I imagine it very probable that a horse, having had a worm in the eye from a relaxed system, will also be very subject to weakness in the loins. This does not argue any particular connexion between these complaints, or that one is in consequence of the other; it only advances that the same habitual or remote cause may produce both. This is, however, entirely hypothesis, which I have presumed to venture, and which, at all events, I conceive much more probable than that extracting the worm from the eye occasions a weakness in the loins." (P. 340, 341.)

Such being the facts of the case, the author endeavours to explain them by the following hypothesis:—It being admitted that the *Strongylus armatus* and *Filiaris papillosa*, frequently observed in the eye of the horse, exist also in the cellular tissue of that animal, especially in the neighbourhood of the lumbar vertebræ, as well as being found in the blood, it is not improbable that, in the course of the circulation, they may attach themselves and multiply in the lumbar region, constituting the principal disease; and that the worm in the eye is casually deposited in the anterior chamber, by means of the circulation of the blood through the large arteries of the ciliary processes. The worm in the eye may be, therefore, only the index of the disease in the lumbar region. Both these worms may often exist in the cellular tissue of the horse, without a single one appearing in the eye; and it may happen that the worm in the eye may be manifested immediately after the generation of the animalculæ in the lumbar region, and before the health of the animal has begun to suffer. It by no means follows, when these worms exist in the loins, that a worm in the eye must necessarily be met with.

It is proper to observe, that this explanation of the invasion of the disease in the horse, is really belonging to Mr. Twining, the author of the second paper, and that Mr. Breton only adopted these suggestions in consequence of reading that paper. In other respects, Mr. Twining's Essay confirms the facts and statements in the first paper.

In concluding our notice of this volume, we must apologise for omitting to mention several of the communications; but we have been chiefly induced to select those subjects, either of the most general interest or which are least known in this country; and, above all, we have been careful to abstain from making remarks of our own, both because many of the subjects are new to us, and because we wished to devote as much space as possible to the authors themselves.

---

*Art. II.—*An Introduction to the Use of the Stethoscope; with its Application to the Diagnosis in Diseases of the Thoracic Viscera: including the Pathology of these various Affections.* By William Stokes, M.D.—12mo. pp. xiii. 226. Edinburgh: Maclachlan and Stewart, 1825.

[Concluded from page 333.]

In our last Number, we laid before our readers a full account of the application of the stethoscope to the diagnosis of pulmonary diseases; on the present occasion we have to direct their attention to the assistance which may be derived from it in affections of the heart. In using it for this purpose, it is necessary to take care that the patient is under no mental or physical excitement: the very act of applying the instrument, for example, would in many produce such an effect on the action of the heart, as to mislead an inexperienced person. We ought, likewise, to avoid making the examination soon after any meal, as in most the pulse is accelerated at such times, and in many it is rendered very frequent, or even irregular.

The phenomena connected with the heart are divided into natural and pathological; an arrangement which we shall follow, beginning with the former.

"*Extent of the Pulsations.*—In a healthy man, whose heart is well proportioned, the pulsations are only heard between the fifth and seventh ribs on the left side, and under the inferior part of the sternum. The motions of the left cavities are heard particularly in the first, those of the right in the second situation. When the sternum is short, the pulsations may be heard in the epigastrium.

"In fat subjects, in whom the pulsations of the heart cannot be felt with the hand, the space in which we can hear them by means of the

* We request our readers to correct an error in the former part of this Review, as it interferes with the sense: instead of "we find it defined to be meaning," read "we find it to mean."—Reviewer.

stethoscope, is sometimes limited to a surface of not more than a square inch.

"In meagre persons, on the contrary, whose chests are ill developed, we distinguish the pulsations over three-fourths of the sternum, from below upwards, sometimes even over the whole of this bone; over the superior part of the chest; and even under the right clavicle. In these cases, when the pulsation is less under the clavicles than in the precordial region, we may conclude that the heart is in good proportion.

"*The shock or impulse.*—By the impulse, is meant the feeling of elevation or percussion which is given by the pulsation of the heart. It is distinct under the stethoscope, when the hand, applied over the precordial region, can feel nothing. In a healthy man, especially if he is moderately fat, it is very little marked. We can distinguish it generally in the precordial region, and the inferior half of the sternum, and always with greater force between the cartilages of the fifth and seventh ribs, the point corresponding to the apex of the heart. Its force varies *ad infinitum* according to the constitution of the subject. Custom teaches us to distinguish when this force is greater or less than in the state of health.

"*The sound.*—In the state of health, the alternate contractions of the different parts of the heart give rise to sounds, easily perceivable by the stethoscope, whatever may be the size and force of the circulatory organ.

"Each pulsation of the heart corresponds to two successive sounds; the one, clear, distinct, and analogous to that produced by the valve of a bellows, corresponds to the systole of the auricles; the other, more dull and prolonged, coincides with the arterial pulsation, and the feeling of impulse mentioned above. It is produced by the contraction of the ventricles.

"The sound of the right cavities is heard in the inferior part of the sternum, that of the left between the fifth and sixth costal cartilages. It is always stronger in the precordial region than in the other parts of the chest, where it may be distinct in subjects in whom the parietes of the heart are thin.

"In these latter cases, the sound of the auricles is more distinct under the clavicles than that of the ventricles, which does not occur at the precordial region.

"In individuals in whom the anterior edges of the lungs are prolonged before the pericardium, the sound of the auricles is more obscure than that of the ventricles. In some cases the sound is no longer distinct.

"*The rhythme.*—By this term we understand the order in which the different parts of the heart contract, and the respective duration and succession of these contractions to one another. When the finger is applied to the pulse of a healthy man at the moment of its diastole, the ear applied to the stethoscope is slightly raised by a motion of the heart synchronous with that of the artery, while at the same time there is a dull sound: this indicates the contraction of the ventricles. Immediately after, and without any interval, a sound more distinct, and of shorter duration, announces the contraction of the auricle: no motion, sensible to the ear, accompanies this sound. An interval of short but well-marked repose succeeds, after which a new contraction of the heart is heard.

"The respective durations of the contractions of the auricles and ventricles, appear to be pretty exactly determined in the following manner:—Of the whole time in which a complete contraction and interval of repose takes place, from a third to a fourth is taken up by the systole of the auricles, a little less than a fourth by absolute repose, and the remainder by the contraction of the ventricles. These relations exist, whatever may be the velocity or frequency of the motions, when the organ is healthy and in good proportion." (P. 136—139.)

The pathological phenomena are divided into those which relate to the extent over which the pulsations can be felt,—to the force of the pulsation, and the nature or intensity of the sound thus produced.

The extent of the heart's pulsation may be either increased or diminished; the augmentation usually proceeds in the following manner:—First, it occurs over the whole of the left side, at its anterior parts; next over the right side; thirdly, over the left side, at its posterior surface; and, fourthly, over the posterior part of the right side, which last, however, is of rare occurrence. The possibility of hearing the pulsations of the heart in these different situations, indicates, for the most part, debility of the organ, as thinness of its parietes or passive dilatation. It may, however, be produced by causes of a temporary nature, as emaciation, nervous agitation, &c.; or from circumstances foreign to the heart itself, as contraction of the chest, induration of the lungs, &c.

*Dilatation of the heart.*—In this, which constitutes the passive aneurism of Corvisart, the cavity of the ventricles is increased in size, the parietes at the same time becoming thin; the muscular substance is softened, and we have seen this proceed to such an extent that it could be torn or broken down by the fingers, like a piece of thick brown paper wetted.

"In this affection, the sound of the pulsations is clearer than natural in the precordial region. The ventricular contraction is distinct and sonorous; the extent in which we can hear the pulsations is increased, while the impulse is trifling.

"This disease may affect both ventricles; it is, however, more frequently met with in the right than the left. The part where the sharp distinct sound is heard, points out the cavity affected: thus, when it is heard between the fifth and seventh ribs on the left side, corresponding to the ventricular contraction, it forms the pathognomonic sign of dilatation of the left ventricle. The same sound heard under the sternum, or between the cartilages of the fifth and seventh ribs on the right side, indicates the dilatation of the right ventricle.

"CASE.—A man of a lymphatic temperament, aged thirty-one, entered the Hospital Cochin, on the 30th July, 1822, stating that, for the last six months, he had been several times attacked with severe colds, accompanied by hæmoptysis. He had now a cough, with abundant expectoration of a thick greenish mucus, not streaked with blood. Dyspnœa on the slightest exertion. Pectoriloquism over almost the whole right side of the chest, with a gurgling rale. Impulse of the heart very feeble; the sound of the ventricles was remarkably clear, almost similar to that obtained by striking on a tambourine; it was heard even at the posterior part of the chest, and only differed from that of the auricles in being a little more prolonged. Pulse small, feeble, and frequent.—Diagnosis: *Dilatation of the ventricles, with thinning of their parietes.*

"He died on the 17th of August, and the body was opened eighteen hours after death.

"Heart enlarged; its parietes soft, flabby, and thin. The right ventricle was not much enlarged, but the left greatly increased in size; and this dilatation must have taken place at the expence of the muscular substance, as the thickness of the left ventricle was hardly greater than that of the right. The auricles were not much altered, either in size or thickness; their valves were of a violet-reddish colour. The right lung presented many tubercular excavations, and was covered by a false membrane of considerable thickness towards the superior part. The left lung posteriorly was infiltrated with serum, and contained miliary tubercles, with a few tubercular excavations." (P. 141—144.)

Query: In the diagnosis of this case, why were the "many tuberculous excavations" in the right lung, and the infiltration of the serum, the presence of miliary tubercles, and the "few tubercular excavations" of the left, omitted?

*Dilatation with hypertrophia.*—In this disease, the cavities and parietes of the heart become simultaneously augmented; the muscular fibres become firmer than natural, and the apex of the heart becomes thicker.

"This alteration, which constitutes the active aneurism of Corvisart, is detected by the strong and extended pulsation in the region of the heart, which appears to repel the hand with force; by the hardness and volume of the arterial pulsations, which may be compared to a column of mercury striking the finger, and which are evident to the sight in many parts of the body. Percussion on the region of the heart gives a dull sound. The ear, on the stethoscope being applied over the heart, receives a strong impulse at each contraction of the ventricles, while a distinct sound is heard. The auricular contractions are sonorous; and the pulsations are heard over a great extent, while their rhythme is rarely altered." (P. 144.)

"Dilatation of the auricles is a case rarely met with, especially when we consider the comparative frequency of a similar affection of the ventricles. It is sometimes found to occur in patients suffering under hypertrophia, or dilatation of the ventricles; but most frequently this does not occur. More rarely the auricles are found dilated, while the ventricles remain in their natural state.

"Dilatation of the auricles may depend on two different causes: first, upon an augmentation of their cavities, which takes place during life, and depends on hypertrophia of one of the ventricles, on ossification, or thickening of the auriculo-ventricular valves. Secondly, upon an apparent increase of size, which takes place during the last moments of life, from the accumulation of blood in the auricles, which become distended on account of the elasticity of their tissue, but return to their original dimensions when the distending cause is removed. In this case they are thin, and allow the colour of the blood to be seen through many points of their surface; while in the former there is generally some thickening of their parietes, and, upon being emptied through their own vessels, the auricles do not regain their original dimensions, but remain permanently enlarged.

"The signs of the auricular dilatation, obtained by means of the stethoscope, may be confounded with those of the alterations of the heart, to which the dilatation is owing. Thus, when the left auricle is affected, the signs obtained may be confounded with those of ossification of the mitral valve; and dilatation of the right auricle is difficult to be distinguished from hypertrophia of the ventricle on the same side. Nevertheless, it may be said that, whenever the auricles are dilated, the sound of their contractions loses the clear and distinct character peculiar to it in the state of health; becomes duller, and analogous to that produced by air suddenly issuing from a bellows.

"The diminution of extent in which the pulsations of the heart can be heard, is generally indicative of a thickening of the parietes of this organ, accompanied by diminution of its capacity. It occurs in softening of the heart, but most commonly in pure hypertrophia. (P. 148, 149.)

*Hypertrophia.*—When the left ventricle is affected with this, its parietes become thickened, the columnæ carneæ, and the inter-ventricular septum, participating in the disease; the cavity of the ventricle diminishing in proportion as its walls are thickened. When the right ventricle is the seat of this disease, the thickening is more uniform, and is said never to exceed four or five lines.

"When the left ventricle is in a state of hypertrophia, its contraction gives a stronger impulse and duller sound than in the natural state. The sound is prolonged in proportion to the degree of hypertrophia, and the contraction of the auricle is very indistinct. The pulsations of the heart are heard over a small extent, sometimes only between the cartilages of the fifth and seventh ribs.

"When the right ventricle is affected, the sound of its contractions is the same as in the former case, except that it is not quite so dull; the impulse is stronger at the inferior part of the sternum than between the cartilages of the fifth and seventh ribs on the left side, which is the contrary of what happens when the left ventricle is diseased.

"When hypertrophia occurs in both ventricles, the symptoms met with are composed of those resulting from the affection of each, but generally those of the right side are predominant. (P. 150.)

*Softening of the heart.*—In this disease the muscular fibres become so soft, that they may be easily torn; the colour varies, —sometimes it is violet, more commonly of a pale yellow; in other instances it is whitish, the substance being flabby, but not friable.

"The sound produced by the contractions of the heart, we have seen to be clearer in dilatation, and duller in hypertrophia. There is a disease of the heart, termed by the French authors *ramollissement*, which consists in a softening of its muscular substance; and in which the sound is duller than natural, and in some cases disappears altogether.

"When, with little impulse, both the contractions of the heart give an equally moderate, dull, and obtuse sound, we may suspect that the heart is softened, but still in good proportion. When this affection is combined with dilatation, the sound, though still clear to a certain degree, is, however, duller than when the latter disease occurs by itself. When it coexists with hypertrophia, the sound of the ventricular contraction can hardly be heard, and in extreme cases disappears completely." (P. 154, 155.)

*Pericarditis.*—The stethoscope has little to boast of in this complaint.

"The symptoms of this affection are very obscure. Laennec himself has confessed that, even with the aid of the stethoscope, it is difficult of detection. M. Collin states that, in some cases of the acute species, he thinks there is heard a sound similar to that of the crackling of new leather, which he attributes to the dryness of the serous membrane, the first consequence of its inflammation. A similar dryness takes place in inflammation of the conjunctiva and the synovial membranes.

"In chronic pericarditis, accompanied by effusion, the pulsations of the heart are obscure, tumultuous, and felt over a great extent of surface." (Pp. 156, 157.)

*Disease of the valves.*—It is well known that ossification, or a state approaching to it, is much more frequently met with in the mitral valves and those of the aorta, than in the bicuspid or those of the pulmonary artery. The valves, indeed, are seldom perfectly ossified, more frequently presenting a cartilaginous appearance, with osseous particles; and this is generally more conspicuous at the apex than at the base. By this thickening of the parts, the aperture is occasionally very much contracted. The growth of warty excrescences may take place either on the valves themselves, or on the inner parietes of the heart: in the former case, they very much resemble venereal warts.

"But there are certain sounds, not heard in the natural state, which M. Laennec first described as accompanying disease of the valves, or narrowing of the orifices of the heart; these are two in number, namely, what the above author terms ***bruit de rape***, and ***bruit de soufflet***; the first analogous to that produced by a saw, and the second to that of a bellows.

"The first of these sounds is considered by M. Laennec to be indicative of ossification of the valves, their cartilaginous induration, or the growth of warty excrescences on them.

"When the mitral valve is affected, the sound of the auricular contraction is more prolonged, duller, and accompanied by a grating noise, which resembles that produced by a saw when drawn over a piece of wood. When the hand is placed over the heart, a vibratory sensation is perceived.

"When the tricuspid valves are affected, the sound is heard on the right side, while it is distinguished from that produced when the sigmoid valves are thus diseased, by the sound in the latter case being isochronous with the contraction of the ventricles, which is more or less prolonged.

"The cause of the second species of sound is still involved in great obscurity. This phenomenon appears to accompany contractions of different parts of the heart or arteries, but may, however, be produced in some individuals, especially those of a nervous temperament, without any alteration in the functions or structure of the heart. It is heard whenever we compress an artery, and listen to its pulsations by means of the stethoscope; also before hemorrhages, in the vessels that carry blood to the part where the bleeding takes place; and generally in palpitations, arising from any cause whatsoever. It is of a peculiarly inconstant character, appearing and disappearing in a short space of time; and may thus frequently mislead an inexperienced observer." (Pp. 160, 161.)

With the following observations on the application of the stethoscope to *aneurism of the aorta*, we terminate our quotations:—

"If, on applying the stethoscope upon the right superior part of the sternum, or under the right clavicle, we hear pulsations, and perceive an impulse isochronous with the pulse, much stronger than that of the ventricles in the precordial region, we may suspect that the ascending aorta or its arch is dilated, as it is extremely rare to find, even in hypertrophia, that the impulse is perceived beyond the precordial region. If this phenomenon is found constant after many examinations, it may be looked upon as affording a sure diagnosis of aneurism. In these severe cases, the pulsations are heard in the superior dorsal region.

"Aneurism of the abdominal aorta may be easily recognised by means of the stethoscope. In this case we hear the pulsations with a degree of intensity which could not be expected from the application of the hand over the affected part. The pulsations are simple; for the contraction of the auricles is not heard, even when the aneurism is above the trunk of the cœliac artery. The sound is clear and distinct, like that of the auricles, but is much stronger." (P. 167.)

In concluding this subject, we would remark, that the greatest misfortune that can befal any individual whose conduct is subjected to inquiry, is the unmeasured praise of injudicious friends;—the surest means of sinking a remedy below its level, is to laud it above its merits, and so we suspect with regard to the stethoscope—its warmest admirers are like to prove its greatest enemies. That it is an instrument capable of some useful applications, such as may prove of occasional assistance, when employed in conjunction with, and subordinate to, that examination of the symptoms more usually adopted, we readily admit. But, when we see it placed before all these,—when we witness a practitioner judging of the presence of inflammation in the lungs, not by any reference to the cough, the pain, or the pulse, but solely from certain sounds emitted by this little instrument of mighty power, we cannot but smile at the infatuation, and lament that what might be of real use, if employed in moderation, is likely to prove worse than useless, through the enthusiasm of its patrons. We earnestly recommend to our readers to regard the stethoscopic examination as secondary only to those indications of disease manifested by symptoms; and, in cases of doubt, to distrust its infallibility, particularly where any practical question is involved. Used thus temperately, it will add one more to the various means we already possess of ascertaining the healthy or morbid condition of the thoracic viscera, and, as a guide to a practical acquaintance with the instrument, we recommend the work of Dr. STOKES.

Good

---

### DIVISION II.

### FOREIGN.

ART. III.—***Precis Elementaire de Physiologie.*** Par M. MAGENDIE, &c. &c. Second Edition.—Paris, 1825.

***An Elementary Compendium of Physiology; for the Use of Students.*** By F. MAGENDIE, M.D. &c. &c. *Translated from the French, with copious Notes, Tables, and Illustrations.* By E. MILLIGAN, M.D. Fellow of the Royal College of Physicians, of the Society of Scottish Antiquaries, Corresponding Member of the Literary and Philosophical Societies of Manchester and Leeds, Extraordinary Member of the Royal Medical Society, and Lecturer on Physiology and Therapeutics, Edinburgh. Second Edition, greatly enlarged.—8vo. pp. 618. Edinburgh, 1826.

ON a recent occasion we introduced to the notice of our readers the great work of RODOLPHI on Physiology, and we now beg to call their attention to the "Elementary Compendium" of M. MAGENDIE, on the same subject. The importance, indeed, of physiology is too obvious to require elucidation; and, to assert its interest and utility, is sufficient to command the assent of our brethren. Yet we fear that too many content themselves with this acknowledgment, and look upon investigations of this nature with some degree of apathy, if not a little distrust. Probably, it is this latter feeling, more than any other, which has tended to prevent this most extensive and most important branch of science from taking the rank among medical studies to which it is unquestionably entitled. Nor, indeed, can we wonder at the scepticism with which many view the doctrines of physiology, when we consider the disposition on the part of most, if not all, writers upon the subject to go beyond their legitimate boundary,—to substitute imagination for reasoning, conjectures for facts, and words for ideas. Surely these remarks are not too severe, nor the censure more than is merited by those systems, in which *organic sensibility*, *nervous fluid*, *vital force*, and such like metaphorical and unintelligible expressions, betray the ignorance they are intended to disguise.

Opposed to these, which we may call the metaphysical systems of physiology, stands the author of the present work, and his views present a striking example of the tendency which reformers have to run into extremes. We mean that M. Magendie, in our opinion, regards too much the multiplying of experiments, and too little the conclusions to be drawn from them. Each new experiment is allowed to constitute a basis for its own little superstructure; so that the science forms an immense collection of detached facts, rather than a comprehensive whole. This strikes us as a considerable objection to the work before us; lessening its interest, at least, though not its accuracy. True it is that, in the present state of our knowledge, it may not be possible to reduce the materials to consistency and order, discarding what is useless, and fixing our attention upon what is really valuable. Yet he who would attempt this task would do more for the advancement of physiology than the most industrious among those who vary their experiments in a thousand ways, without object and without end. Such, we confess, appear to us to be the besetting sin of the French at present, among whom there is a set of young aspirants after fame, who seem only to live *pour faire les experiences.*

At the head of those who cultivate the science in this way, but greatly above his followers, stands MAGENDIE, one of the most industrious and dextrous experimentalists who has ever devoted himself to physiological pursuits. That he thinks more highly of the present mode of cultivating the science than we do, may readily be supposed. "Already (says he) the systems founded upon the organic functions, are no longer received with the same degree of favour; and, in order to publish a work of *romantic physiology*, it is now necessary to make, or to say that one has made, experiments.

"The mischievous and absurd prejudice, that physical laws have no influence upon living bodies, has no longer the same authority; intelligent persons begin to perceive that a great variety of phenomena may exist in living animals, and that actions simply mechanical by no means exclude actions simply vital. We shall hope that physiologists will no longer be proud to boast of their ignorance of the first principles of natural philosophy and chemistry, and of affording deplorable proofs of that ignorance in their works.

"It is now no longer doubtful that researches upon animal bodies may be applied, with admirable precision, to the phenomena of the life of man; the luminous clearness which the recent experiments relative to the nervous functions have thrown upon pathology, removes all uncertainty in that respect: but what proves, much better than I can express, how much the utility of physiological experiments is now felt, is the great number of persons applying themselves to researches of this kind; and the rapidity with which new, unexpected, and important discoveries, succeed each other for some time, and make of the science of life an entirely new system.

"But a few years, and physiology, intimately connected with the physical sciences, shall not be able to make a step without their succour: she will acquire the rigour of their method, the precision of their language, and the certainty of their results: by raising herself thus, it will soon be out of the reach of that ignorant rabble, which, blaming without ever comprehending, is always present and frequent when the object is to oppose the progress of science. Medicine, which is only *the physiology of the sick man*, will soon follow in the same path,—soon reach the same height; and we shall then see all those degrading *systems* which have disfigured it for so long a period, rapidly disappear." (P. xviii. xix.)

The first edition of the work before us appeared some years ago; and an account of it was soon after laid before the readers of this Journal. The present edition, however, demands that we should take some notice of it, as it contains many important alterations and additions, embracing discussions on the recent discoveries in the nervous system, and many new facts concerning the blood, respiration, animal heat, motion, imbibition, and absorption, deduced from the labours of BELL, ROLANDO, and FLOURENS; EDWARDS, PREVOST, and DUMAS; DESMOULINS, DULONG, GASPERD, SEGALAS, and various others. The principal interest and importance of the work, however, depend upon its containing a condensed and perspicuous account of the various discoveries and physiological doctrines of the distinguished author himself.

We have now to say a few words respecting Dr. MILLIGAN's part—the English dress in which he has presented to us the Compendium of M. MAGENDIE. This is scarcely elegant; but it is plain, and the meaning of the author seems faithfully rendered,—a sovereign quality in a translation. We were inclined, indeed, to be somewhat more critical; but we find, on turning to the Preface, that Dr. M. deprecates censure, apologising (as it were) by anticipation. "The translator has long thought that much of the obscurity and *ennui* of physiological discussion arises from that obscure polysyllabick dialect in which it has hitherto been couched; and, to avoid this inconvenience, he has studied plainness of language, almost to rusticity." Now, we must crave permission to differ from Dr. Milligan on this point. That "obscurity" may, or rather must, have resulted from adopting "obscure" dialect, is very obvious; and he has therefore done well to avoid these, as well as the inflated and "polysyllabick" language too often employed to cover the lack of clear and precise ideas: but we cannot imagine in what manner obscurity is to be diminished, or ennui avoided, by the substitution of language plain *almost to rusticity*. Fidelity, however, we repeat, is the first recommendation of every translation; and to this praise we believe the one before us to be fully entitled.

The labours of Dr. Milligan, however, have not been confined to the mere drudgery of rendering his author into English; he has added, in the form of Appendix, copious and valuable notes, in which we find discussions on the Tissues of BICHAT, with tables; on the doctrine of a Double Life; on the Secretions; the Anatomy of the Eye; the Yellow Spot of SOEMMERING; the discoveries of KNOX; the Ciliary Processes; the Canal of PETIT; on the Voice; Muscular Contraction; Respiration; Absorption; Sympathy; Sleep; Craniology, — —, the last being somewhat too much of a controversial nature.

To give any account of the contents of this volume, extensive as the subject is, would occupy much more space than we can devote to; while its elementary nature would render it uninteresting. Our object has been to direct the attention of our readers towards it, and we can confidently recommend it as the most extensive collection of physiological facts which has recently been published.

